# P02.185. The effects of tactile massage (TM) on blood pressure, heart rate and blood glucose in a sample of women suffering from primary insomnia

**DOI:** 10.1186/1472-6882-12-S1-P241

**Published:** 2012-06-12

**Authors:** M Sjöling, M Jong, K Ljadas, E Englund, J Appelberg

**Affiliations:** 1 Mid Sweden University, Sundsvall, Sweden; 2 Västernorrland County Council, Sundsvall, Sweden

## Purpose

The overall objective of this pilot study was to study the direct effects of tactile massage (TM) on blood pressure, heart rate and blood glucose in a sample of women suffering from primary insomnia.

## Methods

The study had an experimental prospective design, with a total of 10 women (mean age; 53 years, ±5.4). The participants underwent TM twice a week for six weeks resulting in a total of 120 treatments. For short-term effects, systolic and diastolic blood pressure, heart rate and blood glucoses were assessed by the therapist before and after each treatment. Long-term assessments were made at baseline, week 7, and week 13.

## Results

As a short term result after the treatment with TM, the participants reached a statistically significant reduction of systolic blood pressure (-5.5 mmHg, ± 5.0), diastolic blood pressure (-2.0 mmHg, ± 4.4), heart rate (-5.1 beats per minute, ± 3.4) and blood glucose (-0.2 mmol, ± 0.5). No long-term effects with respect to the studied variables can be observed.

## Conclusion

In summary, we have shown in a normotensive but highly stressed sample of women, that TM has beneficiary effects on parameters of stress and cardiovascular function. In total, 120 TM treatments were analyzed with respect to the objective of the study, but in order to more understand the practical effects, and to more deeply evaluate TM’s place in the modalities of stress reduction, we recommend further studies with larger samples.

